# Vibratile Cells in the Bronchial Mucous Membrane

**Published:** 1853-01

**Authors:** M. Biermer


From the Provincial Medical and Surgical Jour.

*Vibratile Cells in the Bronchial Mucous Membrane of Man, by* M. BIERMER.

ONE of the most curious revelations of the microscope is the existence of epithelial cells, the free surface of which is covered by extremely fine ciliæ, presenting, during life and immediately after death, a vibratile motion. M. Biermer has seen this curious phenomenon in the bronchial membrane of a man dead of phthisis; and by sprinkling the face with charcoal, was enabled to see that the molecules were uniformly moved from below upwards, proving that the epithelial ciliæ moved in that direction.

